# Omega-3 Fatty Acids Attenuate Neuropathic Pain by Modulating Ferroptotic Stress, Selenoamino Acid Metabolism, and Lipid Remodeling

**DOI:** 10.3390/antiox15070852

**Published:** 2026-07-06

**Authors:** Viet H. Dinh, Magda Descorbeth, Francis Zamora, Jo-Wen Liu, Cono Badalamenti, Salvador Soriano, Johnny D. Figueroa, Marino De León, Alfonso M. Durán

**Affiliations:** 1 Center for Health Disparities and Molecular Medicine, Department of Basic Sciences, Loma Linda University School of Medicine, Loma Linda, CA 92350, USA; vdinh@llu.edu (V.H.D.); magdadescorbeth@llu.edu (M.D.); fczamora@students.llu.edu (F.Z.); joliu@llu.edu (J.-W.L.); jfigueroa@llu.edu (J.D.F.);; 2 Blue Zones^™^ Health, Riverside, CA 92506, USA; cono.badalamenti@bluezoneshealth.com; 3 Department of Pathology and Human Anatomy, Loma Linda University School of Medicine, Loma Linda, CA 92350, USA; ssoriano@llu.edu

**Keywords:** neuropathic pain, omega-3 fatty acids, ferroptosis, GPX4, selenoamino acid metabolism, lipidomics, dorsal root ganglion, chronic constriction injury, painful diabetic neuropathy, docosahexaenoic acid

## Abstract

Neuropathic pain (NP) arises from diverse conditions, including peripheral nerve injury, spinal cord injury (SCI), and painful diabetic neuropathy, yet these disorders share oxidative stress, mitochondrial dysfunction, lipid dysregulation, and altered neuronal excitability. We investigated whether dietary omega-3 polyunsaturated fatty acids modulate ferroptotic stress-associated pathways, defined as lipid peroxidation susceptibility and impaired antioxidant defense rather than overt ferroptotic cell death. Female Sprague–Dawley rats received either a soy oil control diet (SOD) or fish oil omega-3-enriched diet (FOD) before chronic constriction injury (CCI). Behavioral outcomes were assessed using Hargreaves and CatWalk testing, followed by dorsal root ganglion (DRG) RNA sequencing, RT-PCR, and GPX4 ELISA. Previously generated SCI metabolomics and human diabetic serum metabolomic/lipidomic datasets were re-analyzed for shared pathways. FOD attenuated CCI-induced thermal hypersensitivity and improved gait parameters. DRG transcriptomics showed reduced injury-associated transcriptional disruption, enrichment of selenoamino acid metabolism, nonsense-mediated decay, and ribosomal quality-control pathways, and reduced mitochondrial dysfunction pathway activity. Omega-3 increased Gpx1/Gpx4 expression and GPX4 protein, reduced pain-associated genes including Scn10a, Piezo2, Trpa1, and Oprm1, and aligned with selenoamino acid enrichment in SCI and human datasets. Human lipidomics showed MG/DG/PC/PE pathway remodeling. These findings support ferroptotic stress as a plausible shared downstream mechanism modulated by omega-3 supplementation across NP models.

## 1. Introduction

Neuropathic pain (NP) is a debilitating condition arising from injury or disease of the somatosensory nervous system and encompasses both peripheral and central etiologies. Clinically, NP manifests as spontaneous pain, hyperalgesia, and allodynia, resulting in substantial functional impairment and reduced quality of life [1,2]. Epidemiological studies estimate that NP affects approximately 7–10% of the global population, with markedly higher prevalence in metabolic disorders such as type 2 diabetes mellitus (T2DM) [3,4]. Painful diabetic peripheral neuropathy (pDN), one of the most common forms of NP, affects 20–50% of individuals with T2DM and represents a major contributor to morbidity and healthcare burden [5,6,7]. Despite its prevalence, current pharmacological therapies provide only partial symptomatic relief and fail to address the underlying disease mechanisms [1,7], underscoring the need for mechanistically informed therapeutic strategies. NP is also a frequent consequence of traumatic nerve injury. Traumatic peripheral nerve injuries occur at an estimated incidence of approximately 14 per 100,000 persons per year, and chronic NP develops in a substantial proportion of affected patients, with reported prevalence exceeding 50% after major nerve injury. Spinal cord injury (SCI) carries an even greater burden: systematic review and meta-analysis estimate a pooled NP prevalence of approximately 53% following SCI, with below-level pain affecting roughly 27–30% and at-level pain roughly 19–20% of patients. The chronic constriction injury (CCI) and SCI models used here therefore represent two of the most clinically prevalent and therapeutically challenging forms of NP.

The pathophysiology of NP, particularly in pDN, is complex and multifactorial. While hyperglycemia has traditionally been considered the primary driver of nerve injury, increasing evidence indicates that metabolic dysfunction extends beyond glucose toxicity to include lipid dysregulation, mitochondrial impairment, and oxidative stress [7,8]. Excess substrate availability in diabetes promotes mitochondrial overload, reactive oxygen species (ROS) generation, and lipid peroxidation, collectively contributing to neuronal dysfunction and axonal degeneration. Importantly, these processes are not unique to diabetic neuropathy but are also observed in other models of nerve injury, including CCI and SCI [9,10,11], suggesting the presence of shared molecular mechanisms across NP etiologies.

Ferroptosis, a regulated form of cell stress driven by iron-dependent lipid peroxidation and impaired antioxidant defenses, has recently emerged as a potential mechanism linking metabolic dysfunction to neuronal injury and neurodegenerative diseases [9,10,12,13,14,15]. Unlike classical forms of cell death, ferroptosis is fundamentally a redox-dependent process characterized by accumulation of oxidized phospholipids and failure of protective systems such as glutathione and glutathione peroxidase 4 (GPX4). Notably, key features associated with ferroptotic stress, including mitochondrial dysfunction, ROS accumulation, and lipid peroxidation, are consistently observed in pDN, CCI, and SCI models. These observations suggest that ferroptosis-associated oxidative stress may represent a shared pathological axis underlying NP across diverse conditions, rather than a mechanism restricted to a single disease context. Throughout this manuscript, we use the term “ferroptotic stress” to denote the pathway-level signature of lipid-peroxidation susceptibility and impaired antioxidant defense: that is, the upstream biology of ferroptosis, rather than overt ferroptotic cell death. Because GPX4 is a selenoprotein whose biosynthesis depends on selenocysteine incorporation, selenium availability and selenoamino acid metabolism are rate-limiting for ferroptotic stress defense, positioning this pathway as a potentially modifiable node in NP biology. Although the two injury models examined here, CCI and SCI, differ in their initial causes, they converge on several downstream processes that are relevant to omega-3 intervention. In CCI, sciatic nerve ligation leads to focal demyelination, Wallerian degeneration, ectopic afferent firing, neuroinflammation within the dorsal root ganglion (DRG), mitochondrial dysfunction, and heightened oxidative stress. In contrast, SCI initiates a secondary cascade involving ischemia, glutamate excitotoxicity, lipid peroxidation, hemorrhage-induced iron release, and progressive oxidative damage. Despite these distinct origins, both models engage overlapping pathological mechanisms that can be modulated by omega-3 PUFAs. Omega-3s may attenuate neuroinflammation, reduce oxidative stress, support mitochondrial function, alter membrane phospholipid composition and lipid peroxidation susceptibility, and serve as substrates for specialized pro-resolving mediators. This convergence provides a mechanistic rationale for investigating ferroptotic stress pathways as a unifying axis of NP.

Dietary omega-3 polyunsaturated fatty acids (omega-3 PUFAs), particularly docosahexaenoic acid (DHA) and eicosapentaenoic acid (EPA), have emerged as promising modulators of NP due to their neuroprotective properties [16,17,18]. Preclinical studies demonstrate that omega-3 supplementation reduces hyperalgesia, improves nerve function, and attenuates neuroinflammation in models of nerve injury [11]. Clinical studies further support these findings, with omega-3 supplementation improving NP symptoms in individuals with T2DM [16]. These effects are commonly attributed to the anti-inflammatory and antioxidant properties of omega-3 PUFAs, including suppression of pro-inflammatory signaling pathways and enhancement of endogenous antioxidant defenses. However, these mechanisms have largely been studied in isolation, and a unifying redox-based framework explaining their effects on NP remains lacking. A particularly unresolved question is the omega-3 paradox in the context of ferroptosis biology: long-chain PUFAs, including DHA and EPA, are themselves the lipid class most susceptible to peroxidation and the canonical substrates for ferroptotic membrane damage. How a PUFA-class dietary intervention reduces, rather than amplifies, ferroptotic stress is not yet mechanistically explained. We hypothesize that the resolution lies not in fatty acid abundance alone, but in coordinated phospholipid remodeling and induction of selenium-dependent antioxidant capacity, an interpretation that the present integrated analysis is positioned to test.

Our prior work provides important translational support for this framework. We previously demonstrated that omega-3 supplementation in individuals with T2DM reduces NP symptoms and alters circulating sphingolipid profiles associated with lipotoxic stress [16]. Subsequent analyses revealed activation of protective metabolic pathways, including antioxidant and redox-regulating systems [17], and more recently, reductions in neurotoxic lipid mediators alongside enhanced markers of cellular repair and lipid remodeling [19]. Collectively, these findings suggest that lipid remodeling and redox regulation are central to the therapeutic effects of omega-3 PUFAs in NP and implicate ferroptosis-associated pathways as a key mechanistic link.

To our knowledge, no prior study has tested whether ferroptosis-associated pathways represent a shared molecular axis across traumatic and metabolic NP, or whether omega-3 supplementation modulates this axis through selenium-dependent antioxidant defense and phospholipid remodeling. To address this gap, we integrated behavioral, transcriptomic, metabolomic, and lipidomic analyses across preclinical models of peripheral nerve injury and central neurotrauma, including CCI and SCI, together with human diabetic datasets. We found that omega-3 supplementation attenuated NP-related behaviors, increased antioxidant-associated markers linked to selenoamino acid metabolism and GPX4, altered glutamate-handling pathways, and promoted coordinated lipid remodeling. These effects converged on pathways associated with reduced lipid-peroxidation susceptibility and ferroptosis-associated oxidative stress, supporting a model in which ferroptotic stress may represent a shared and targetable vulnerability across NP etiologies. Although prior studies, including our own, have shown that omega-3 supplementation reduces NP behaviors and modulates inflammatory and lipid mediators in individual models, three features distinguish the present work: first, it evaluates ferroptosis-associated biology across mechanistically distinct peripheral, central, and metabolic NP contexts; second, it integrates transcriptomic, metabolomic, and lipidomic datasets within a unified ferroptotic-stress framework; and third, it proposes a testable biochemical explanation for the omega-3 paradox, whereby peroxidation-prone PUFAs may reduce, rather than amplify, ferroptotic stress through PEMT- and CHPT1-associated phospholipid remodeling and selenium-dependent antioxidant induction.

## 2. Materials and Methods

### 2.1. Study Design Overview

This study integrates three complementary datasets: (1) a controlled rat CCI model with behavioral and transcriptomic analyses, (2) re-analysis of previously generated SCI metabolomic datasets, and (3) secondary analysis of human diabetic serum metabolomic and lipidomic datasets following omega-3 supplementation. Each dataset was analyzed independently and subsequently integrated to identify shared molecular pathways associated with omega-3-mediated modulation of NP and ferroptosis-associated oxidative stress.

### 2.2. Animals, Diets, and Study Design

All animal procedures were conducted in accordance with NIH guidelines and approved by the Loma Linda University Health Institutional Animal Care and Use Committee (IACUC; approval no. 20-130). Female Sprague–Dawley rats (8–10 weeks old, 182–212 g; Charles River Laboratories, Portage, MI, USA) were housed individually under a 12 h light/dark cycle with ad libitum access to food and water. Female rats were used to maintain consistency and comparability with our previously published omega-3 SCI studies, whose metabolomic data are re-analyzed here, because painful diabetic neuropathy (pDN) and several chronic pain conditions show higher prevalence in women. Only female animals were studied; sex as a biological variable was therefore not assessed (see Limitations).

Following a 1 week acclimatization period, animals were randomly assigned, using a computer-generated randomization scheme, to either a soy oil control diet (SOD) or a fish oil omega-3-enriched diet (FOD). The SOD was based on the standard AIN-93G formulation. The FOD consisted of a modified AIN-93G formulation in which the lipid component was replaced with fish oil enriched in DHA and EPA, as previously described [11,20]. Both diets were formulated to be isocaloric and matched for macronutrient composition. Detailed diet composition is provided in Supplementary Tables S1 and S2.

After 4 weeks of dietary intervention, animals underwent either CCI or SCI surgery and were subdivided accordingly. For the CCI cohort, experimental groups included control/sham, control/CCI, omega-3/sham, and omega-3/CCI animals. The CCI experiment used a 2 × 2 factorial design (diet × injury) with sham-operated and CCI-injured rats under either control or omega-3 diets. Diets were maintained for an additional 4 weeks post-surgery. Sample sizes (*n* = 6–8 rats per behavioral group and *n* = 3–4 per molecular group) were based on prior studies using similar designs and effect sizes, rather than a formal a priori power calculation. Each individual rat was treated as a single experimental unit for all behavioral and molecular analyses.

Group allocation and diet preparation were performed by investigators who were not involved in behavioral testing or molecular analyses. Surgeons were necessarily aware of injury status but were blinded to diet group. Behavioral testing, molecular assays, and statistical analyses were conducted with investigators blinded to diet and injury assignments until all primary analyses were completed.

Animals were excluded only if predefined technical criteria were not met, including incomplete injury parameters, incomplete paw placement during gait analysis, or failure to meet prespecified behavioral inclusion thresholds. No exclusions were made based on post hoc outcome differences. These inclusion and exclusion criteria were defined a priori before data collection.

Behavioral testing was performed in a rotating order across groups to avoid systematic time-of-day or sequence effects. Cages were distributed across shelves and racks without clustering by diet or injury group. No additional randomization or blocking factors were applied.

The primary behavioral outcomes prespecified for hypothesis testing and sample-size considerations were the Hargreaves withdrawal latency ratio and CatWalk gait parameters at 1–4 weeks post-CCI. Molecular readouts (DRG transcriptomics, qPCR, and GPX4 ELISA) were considered secondary outcomes.

### 2.3. Chronic Constriction Injury Surgery

CCI or sham surgery was performed as previously described [21]. Animals were anesthetized with 5% isoflurane for induction and maintained at 2% isoflurane via facemask. The left sciatic nerve was exposed through a 3–4 mm incision below the femur. Four loosely tied chromic gut 4-0 ligatures (Ethicon, Somerville, NJ, USA), spaced approximately 1 mm apart, were placed proximal to the trifurcation of the sciatic nerve. Sham animals underwent identical exposure of the sciatic nerve without ligature placement. Wounds were closed with muscle sutures and skin staples, followed by a 1-week recovery period prior to behavioral testing.

### 2.4. Spinal Cord Injury Surgery

SCI or sham procedures were performed at thoracic level T10 using the NYU/MASCIS impactor device. Animals were anesthetized with ketamine (80 mg/kg) and xylazine (10 mg/kg) administered intraperitoneally. Following laminectomy at T10 with intact dura, the spinal cord was subjected to a standardized weight-drop injury using a 10 g rod dropped from a height of 12.5 mm. Sham animals underwent laminectomy without impact injury. Muscle layers were sutured, and skin was closed with surgical clips.

### 2.5. Behavioral Assessments

Thermal hyperalgesia, mechanical allodynia, and gait were evaluated in the CCI cohort at baseline and at 1, 2, 3, and 4 weeks post-surgery. To reduce inter-animal variability and account for time-dependent changes, all behavioral data were normalized to sham controls using the following formula:

$$Normalized\;response\;(\%)\;=\;\left[\frac{\frac{\left(\frac{L_{inj,X}}{R_{inj,X}}\right)}{\left(\frac{L_{sham,X}}{R_{sham,X}}\right)}}{\frac{\left(\frac{L_{inj,BL}}{R_{inj,BL}}\right)}{\left(\frac{L_{sham,BL}}{R_{sham,BL}}\right)}}\right]\;\times \;100$$

where *L* denotes the left (injured-side) hind paw value and *R* denotes the right (contralateral, uninjured-side) hind paw value. Subscripts “inj” and “sham” indicate values measured in CCI-injured and sham animals, respectively, while subscripts “*X*” and “BL” indicate the post-surgical time point and baseline measurement. This double-normalized index expresses the change in ipsilateral-to-contralateral asymmetry in injured animals, adjusted to sham controls, and referenced to baseline. Normalization was applied consistently across behavioral assays prior to statistical analysis.

Thermal hyperalgesia was assessed using the Hargreaves test [22]. Animals were acclimated for 30 min in enclosed compartments before testing. An infrared heat source was applied to the plantar surface of the hind paw, and withdrawal latency was recorded across four trials with 5 min intervals between trials.

Mechanical Allodynia was assessed by measuring the withdrawal threshold of the hind paws in response to a mechanical stimulus using an electronic von Frey aesthesiometer (model 2391C; IITC Life Science, Woodland Hills, CA, USA). Each animal was placed in a Plexiglas chamber positioned on an elevated metallic grid floor, which provided access to the plantar surface of the hind paw. Animals were allowed to acclimate to the environment for 30 min before testing. A rigid blunt tip attached to the meter was applied to the plantar surface from under the floor. The withdrawal threshold was defined as the average force (g) required for paw removal in five trials separated by a 1 min interval. The data were normalized to the percent change from baseline and sham animals.

Gait analysis was performed using the CatWalk XT system with standard acquisition and analysis settings (Supplementary Table S3). Animals traversed a glass walkway, and paw placement parameters were recorded using internally reflected light and high-speed imaging. Only compliant runs, defined as runs within a speed range of 35–85 cm/s, were included in the analysis.

### 2.6. Tissue Collection and Preparation

Animals were deeply anesthetized and transcardially perfused with ice-cold phosphate-buffered saline (PBS). Spinal cord tissue and dorsal root ganglia (DRG) were dissected, flash-frozen in liquid nitrogen, and stored at −80 °C until downstream molecular analyses. Spinal cord tissue was used for metabolomic analysis, while DRG tissue was used for RNA sequencing, quantitative RT-PCR, and protein-level validation. Transcardial PBS perfusion under brief deep anesthesia (<5 min per animal) was used as the standard method to clear blood from tissue prior to molecular analysis; this procedure applies only to the rat DRG transcriptomic, RT-PCR, and protein endpoints. The metabolomic and lipidomic data reported in this study are re-analyses of previously published, independently collected datasets and did not involve this procedure.

### 2.7. Metabolomic Analysis

Untargeted metabolomic profiling of spinal cord tissue was conducted using ultra-high-performance liquid chromatography/tandem mass spectrometry (UHPLC/MS/MS^2^) under both basic and acid-optimized conditions, as well as gas chromatography/mass spectrometry (GC/MS), as previously described [17,19,23]. Metabolites were identified by comparison to reference library entries based on retention time, mass-to-charge ratio (*m*/*z*), and MS/MS spectral data, followed by manual quality control curation.

For pathway-level interpretation, metabolomic data were analyzed to identify biochemical pathways associated with lipid metabolism, oxidative stress, glutathione metabolism, selenoamino acid metabolism, and ferroptosis-associated oxidative stress.

### 2.8. RNA Sequencing and Bioinformatics

RNA was isolated from DRG tissue using the Qiagen AllPrep DNA/RNA/Protein Mini Kit (cat. no. 80004; Qiagen, Hilden, Germany), which enables isolation of RNA, DNA, and protein fractions from the same biological sample. RNA quality and integrity were assessed using the Agilent Bioanalyzer 2100. RNA libraries were prepared using poly-A selection and sequenced on Illumina platforms. Two planned RNA-seq samples (one SOD-CCI, one FOD-CT) were excluded prior to library preparation because total RNA yield fell below the prespecified minimum concentration and integrity thresholds; no other RNA-seq samples were excluded.

Raw FASTQ files were processed using fastp for quality control and adapter trimming, followed by alignment to the reference genome using HISAT2 (v2.0.5). Gene expression was quantified using featureCounts and normalized as fragments per kilobase per million reads (FPKM).

To improve analytical rigor, only genes with a minimum read depth of 10 counts in at least 90% of samples were retained. Differential expression analysis was performed using DESeq2 (v1.44.0) with Benjamini–Hochberg correction. Differentially expressed genes were identified using an adjusted *p*-value threshold of ≤0.1 and an absolute fold-change threshold of ≥1.2. A relaxed threshold was selected to capture pathway-level signals in the context of modest sample size, with key findings validated using RT-PCR and protein-level assays.

Ingenuity Pathway Analysis (IPA; QIAGEN, Redwood City, CA, USA; https://digitalinsights.qiagen.com/products-overview/discovery-insights-portfolio/analysis-and-visualization/qiagen-ipa/, accessed on 5 April 2026) was used for pathway enrichment and upstream regulator analysis using default settings, including z-score activation thresholds and Benjamini–Hochberg correction for pathway enrichment.

### 2.9. Quantitative RT-PCR

Total RNA was reverse-transcribed, and quantitative RT-PCR was performed as previously described with minor modifications [24]. Gene expression was quantified using SYBR Green chemistry on a real-time PCR system. Relative expression levels were calculated using the 2^−ΔΔCT^ method and normalized to β-actin as the housekeeping gene. Primer sequences used for target genes are provided in Supplementary Table S4.

### 2.10. ELISA

GPX4 protein levels in DRG tissue were quantified using a commercially available ELISA kit (AFG Bioscience, catalog no. EK240233) according to the manufacturer’s instructions. DRG tissues were processed using the Qiagen AllPrep DNA/RNA/Protein Mini Kit (Qiagen, cat. no. 80004) to isolate RNA, DNA, and protein fractions from the same biological sample. The protein fraction was collected and used for downstream ELISA analysis.

The total protein concentration was determined using the DC (Detergent Compatible) Protein Assay (Bio-Rad, Hercules, CA, USA). Samples and standards were run in duplicate, and absorbance was measured using a microplate reader at the recommended wavelength. Concentrations were calculated based on standard curves generated using known concentrations of GPX4. GPX4 levels were normalized to total protein concentration and expressed relative to SOD/sham samples. For ELISA quantification, each biological sample was assayed in technical duplicate (the same biological sample loaded into two adjacent wells on the same plate), and the mean of the two technical wells was used as that animal’s value. Biological replicates were the individual animals (*n* = 3–4 per group), and all statistical comparisons were performed on biological replicates rather than technical replicates. As pre-specified quality control, technical duplicates with a coefficient of variation greater than 15% were flagged and re-assayed, and plates with a standard-curve R^2^ below 0.99 were rejected and re-run.

### 2.11. Human Cohort and Omics Data

Human metabolomic and lipidomic data were obtained from the En Balance-Plus study, a previously published longitudinal intervention evaluating omega-3 supplementation in individuals with type 2 diabetes. The current study represents a secondary analysis of previously collected datasets.

The study was approved by the Loma Linda University Institutional Review Board (LLU-IRB# 5110318) and adhered to the Declaration of Helsinki; informed written consent was obtained from all participants.

Forty self-identified Hispanic/Latino adults (aged 33–74 years) with T2DM completed the 3-month intervention. Participants were recruited from the local community and met the inclusion criteria for stable diabetes management without acute complications. Additional demographic and clinical characteristics have been previously described [16].

Participants received daily omega-3 PUFA supplementation, high-DHA, consisting of 1000 mg DHA and 200 mg EPA over a 3-month intervention period. Plasma and serum samples were collected following a 12 h overnight fast at baseline and post-intervention.

Untargeted metabolomic and lipidomic analyses were performed by Metabolon (Durham, NC, USA) using established UHPLC/MS/MS and GC/MS platforms [23,25]. Metabolites were identified through comparison to a reference library based on retention time, mass-to-charge ratio (*m*/*z*), and MS/MS spectral data, followed by quality control curation. A minimum metabolite presence threshold of 70% across samples was applied.

### 2.12. Statistical Analysis

All statistical analyses were performed using GraphPad Prism (v10.6.0; GraphPad Software, Boston, MA, USA; www.graphpad.com), with statistical significance set at *p* < 0.05 unless otherwise specified. All data are presented as the mean ± standard error of the mean (SEM).

Behavioral data from the Hargreaves test, reported as withdrawal latency ratios, and CatWalk gait analysis parameters, including swing duration, stand time, duty cycle, stride length, step cycle, mean intensity, maximum contact mean intensity, maximum contact maximum intensity, and print area ratios, were analyzed to evaluate the effects of diet, injury, and time. Diet groups consisted of omega-3-enriched fish oil diet (FOD) and control soy oil diet (SOD), while injury groups consisted of CCI and sham animals. Behavioral outcomes were compared across baseline and weeks 1–4 post-CCI, with 6–8 rats per group, including SOD-sham, SOD-CCI, FOD-sham, and FOD-CCI groups.

A two-way mixed-effects ANOVA model with Bonferroni post hoc testing was used to account for the repeated-measures design and longitudinal structure of the behavioral data. This model accounts for within-subject correlations across time points and enables assessment of main effects and interactions among diet, injury, and time.

For CatWalk analysis, missing data points arising from incomplete paw placement or equipment-related issues were addressed using mean imputation within the respective experimental group at the corresponding time point, including baseline and weeks 1–4. Mean imputation was selected to preserve sample size in the context of a repeated-measures design and limited cohort size. This approach assumes missingness at random and maintains group-level central tendency, which is appropriate for balanced designs with small sample sizes [26]. Sensitivity analyses, including complete-case and median imputation approaches, yielded consistent results and confirmed that imputation did not significantly alter the findings.

RT-PCR and ELISA data were analyzed using two-way ANOVA followed by Tukey’s post hoc tests to assess differences in gene expression and protein levels across experimental groups.

## 3. Results

### 3.1. Omega-3 Supplementation Attenuates Neuropathic Pain-Related Behavioral Changes Following CCI

To determine whether omega-3 supplementation altered the development of NP-related behavioral deficits, rats were fed either a SOD or FOD before CCI or sham surgery. Behavioral outcomes were assessed longitudinally at baseline and weeks 1–4 post-CCI. Both the SOD and FOD were custom-formulated and manufactured by Bio-Serv (Flemington, NJ, USA).

**Figure 1:**
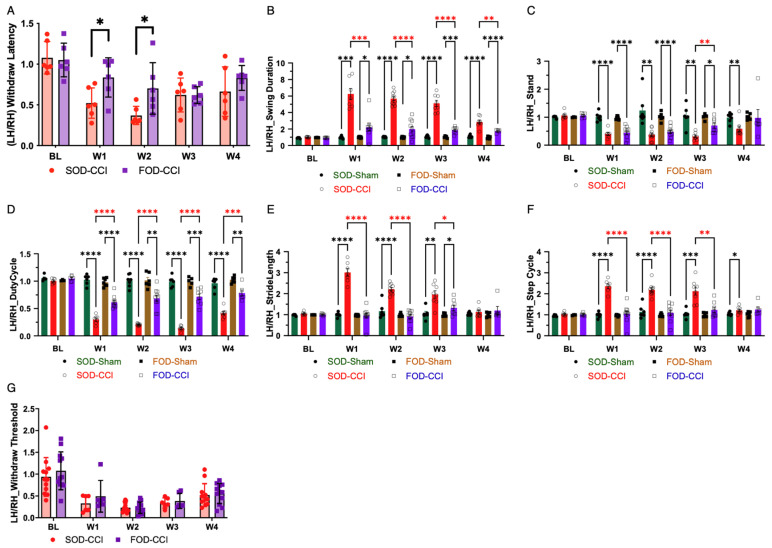
Omega-3 supplementation attenuates NP-related behavioral deficits after CCI. Behavioral outcomes were assessed in rats fed either a SOD or FOD before CCI or sham surgery. Measurements were collected at baseline and weeks 1–4 post-CCI and are presented as normalized injured-to-contralateral hind paw ratios. (**A**) Hargreaves withdrawal latency ratio assessing thermal hypersensitivity. FOD-CCI animals maintained higher withdrawal latency ratios than SOD-CCI animals at early post-injury time points. (**B**–**F**) CatWalk gait parameters assessing locomotor asymmetry and pain-related functional impairment: (**B**) swing duration ratio, (**C**) stand time ratio, (**D**) duty cycle ratio, (**E**) stride length ratio, and (**F**) step cycle ratio. (**G**) Mechanical allodynia was assessed with an electronic von Frey aesthesiometer in CCI rats fed a SOD-CCI or an FOD-CCI. Data show mean ± SEM normalized left/right hind paw withdrawal thresholds at baseline (BL) and weeks 1–4 (W1–W4) post-CCI, with individual values for each animal (SOD-CCI, red circles; FOD-CCI, purple squares; *n* = 12 at BL, W2, and W4; *n* = 6 at W1 and W3). Omega-3 supplementation did not significantly alter mechanical withdrawal thresholds at any post-surgical time point (*p* > 0.05 at all time points), indicating that the behavioral effect of omega-3 supplementation in this model is modality-specific. Data are shown as mean ± SEM; *n* = 6–8 rats per group for the Hargreaves and CatWalk analyses. Behavioral data were analyzed using a two-way mixed-effects ANOVA with Bonferroni post hoc testing. Statistical significance is indicated in each panel by color-coded asterisks: red asterisks denote the SOD-CCI versus FOD-CCI comparison (the primary omega-3 treatment effect), and black asterisks denote all other pairwise comparisons. * *p* < 0.05, ** *p* < 0.01, *** *p* < 0.001, **** *p* < 0.0001.

Thermal hypersensitivity was assessed using the Hargreaves test. Following CCI, SOD-CCI animals showed reduced withdrawal latency ratios compared with sham controls, consistent with increased thermal sensitivity. In contrast, FOD-CCI animals maintained higher withdrawal latency ratios than SOD-CCI animals at early post-injury time points, indicating attenuation of CCI-induced thermal hypersensitivity (Figure 1A).

CatWalk gait analysis was then used to evaluate pain-related locomotor asymmetry and functional impairment. SOD-CCI animals showed altered gait parameters across multiple measures, including swing duration, stand time, duty cycle, stride length, and step cycle ratios. FOD-CCI animals showed improved gait symmetry compared with SOD-CCI animals, with values closer to baseline- or sham-normalized ratios across several parameters (Figure 1B–F). Additional CatWalk parameters evaluating paw pressure and paw-contact characteristics, including mean intensity, maximum contact mean intensity, maximum contact maximum intensity, and print area ratios, showed similar omega-3-associated improvement patterns and are provided in Supplementary Figure S1.

Together, these behavioral data show that omega-3 supplementation attenuated CCI-induced thermal hypersensitivity and improved multiple gait parameters associated with NP-related functional impairment. Mechanical allodynia was assessed in parallel using an electronic von Frey aesthesiometer. Omega-3 supplementation did not significantly alter mechanical withdrawal thresholds in CCI animals at any post-surgical time point compared with control CCI animals (Figure 1G; *p* > 0.05 at all time points). The absence of a significant omega-3 effect on mechanical allodynia, in contrast to the significant attenuation of thermal hypersensitivity and the improvement in gait, indicates that the behavioral effects of omega-3 supplementation in this model are modality-specific. Mechanical allodynia was assessed in parallel using an electronic von Frey aesthesiometer. Omega-3 supplementation did not significantly alter mechanical withdrawal thresholds in CCI animals at any post-surgical time point compared with control CCI animals (Figure 1G; *p* > 0.05 at all time points). The absence of a significant omega-3 effect on mechanical allodynia, in contrast to the significant attenuation of thermal hypersensitivity and the improvement in gait, indicates that the behavioral effects of omega-3 supplementation in this model are modality-specific.

### 3.2. Transcriptomic Profiling of DRG Reveals Omega-3-Associated Changes in Injury-Response, Mitochondrial, Selenoamino Acid, and Translational Quality-Control Pathways Following CCI

To define transcriptional changes associated with the behavioral phenotype, bulk RNA sequencing was performed on ipsilateral and contralateral dorsal root ganglia (DRG) collected 1 week after CCI, a time point selected to capture early injury-evoked transcriptional responses after injury in this NP model. Four groups were analyzed: SOD-CT, SOD-CCI, FOD-CT, and FOD-CCI (*n* = 3–4 biological replicates per group; SOD-CCI and FOD-CT, *n* = 3; SOD-CT and FOD-CCI, *n* = 4). Differentially expressed genes (DEGs) were identified using a minimum fold-change threshold of 1.2 and an adjusted *p* value ≤ 0.10. These relatively permissive thresholds were chosen a priori to increase sensitivity to modest early transcriptional changes in DRG after CCI while controlling the expected false discovery rate in the context of a limited sample size.

**Figure 2:**
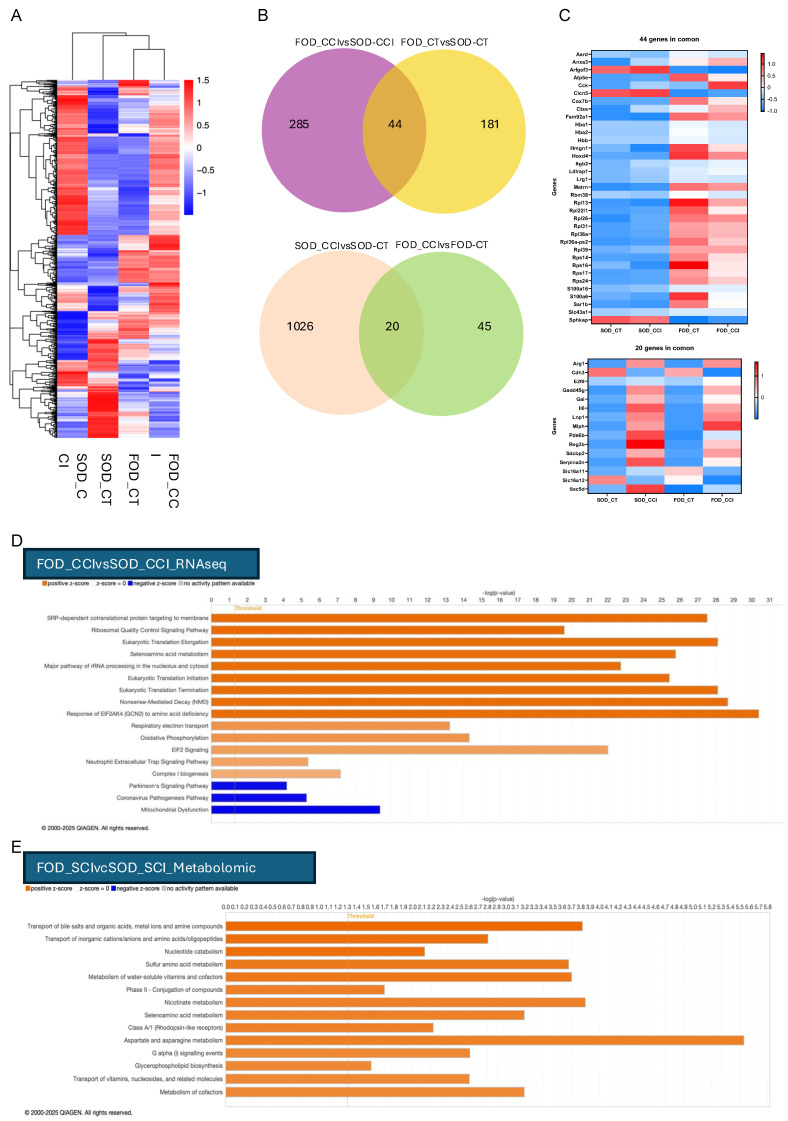
DRG transcriptomic and spinal cord metabolomic analyses identify omega-3-associated modulation of injury-response, mitochondrial, and selenoamino acid pathways. Dorsal root ganglia (DRG) were collected 7 days after CCI from SOD-CT, SOD-CCI, FOD-CT, and FOD-CCI animals and analyzed by RNA sequencing. (**A**) Global heatmap of differentially expressed genes across experimental groups. (**B**) Venn diagram summarizing differentially expressed gene overlaps across pairwise comparisons. SOD-CCI versus SOD-CT showed 1026 unique DEGs, whereas FOD-CCI versus FOD-CT showed 45 unique DEGs, with 20 shared genes. FOD-CCI versus SOD-CCI identified 285 unique DEGs, and FOD-CT versus SOD-CT identified 181 unique DEGs, with 44 shared genes. (**C**) Targeted heatmap of shared differentially expressed genes across comparisons. (**D**) IPA of FOD-CCI versus SOD-CCI DRG RNA-seq data showing enriched canonical pathways, including selenoamino acid metabolism, NMD, ribosomal quality control, and reduced mitochondrial dysfunction pathway activity. (**E**) IPA analysis of spinal cord metabolomic data from FOD-SCI versus SOD-SCI animals showing pathway-level changes, including enrichment of selenoamino acid metabolism and amino acid transport/metabolism pathways. Pathway activation is based on IPA z-score predictions.

The global heatmap of significant DEGs demonstrated clear group-dependent separation of transcriptomic profiles (Figure 2A). The SOD-CCI group showed the largest deviation from contralateral controls, with broad induction of injury-associated transcripts and suppression of multiple metabolic genes. In contrast, the FOD-CCI samples clustered more closely with FOD-CT and sham-like profiles, indicating a more restricted transcriptional response following injury. Among the genes increased in SOD-CCI relative to SOD-CT were inflammatory and injury-responsive transcripts, including C3 (log2FC 2.74, adjusted *p* = 0.016) and Arg1 (log2FC 4.38, adjusted *p* = 9.43 × 10^−6^), whereas genes linked to neuropeptide signaling and homeostatic function, such as Adcyap1, were reduced (log2FC −2.73, adjusted *p* = 1.45 × 10^−38^). In FOD-CCI samples, several of these shifts were attenuated relative to SOD-CCI, and the overall magnitude of transcriptional disruption was reduced.

To further define the contributions of diet and injury, DEG overlap was examined using pairwise comparisons (Figure 2B). Comparison of FOD-CCI versus FOD-CT identified 45 unique DEGs, whereas SOD-CCI versus SOD-CT identified 1026 unique DEGs, with only 20 genes shared between these comparisons. This difference indicates a substantially larger injury-driven transcriptional response in the SOD group than in the omega-3 group. In the diet-only comparisons, FOD-CCI versus SOD-CCI yielded 285 unique DEGs and FOD-CT versus SOD-CT yielded 181 unique DEGs, with 44 genes shared between these comparisons, indicating that dietary omega-3 exposure altered the DRG transcriptome both at baseline and after injury.

A targeted heatmap of common DEGs highlighted transcripts that changed in a consistent direction across comparisons (Figure 2C). These included genes related to oxygen handling, chromatin organization, and immune regulation, such as Hba2 (log2FC 3.41 vs. SOD-CT, adjusted *p* = 1.01 × 10^−6^), Hmgn1 (log2FC 1.00, adjusted *p* = 0.1), and Ssc5d (log2FC −0.61, adjusted *p* = 0.58). Relative to SOD-CCI, the FOD-CCI group showed a more restricted expression pattern across these shared genes, with values shifting toward those observed in contralateral controls.

To determine whether these transcriptional differences converged on specific biological programs, DEGs from the FOD-CCI versus SOD-CCI comparison were analyzed using IPA (Figure 2D). The most significantly inhibited canonical pathway was mitochondrial dysfunction (z-score = −3.441, −log(*p* value) = 9.44), with contributing genes including Atp5f1e, Atp5mf, Atp5mg, and Ndufa5. Among activated pathways, nonsense-mediated decay (NMD) showed a z-score of 5.292 with a −log(*p* value) of 28.8, selenoamino acid metabolism showed an identical z-score of 5.292 with a −log(*p* value) of 25.9 (IPA returns matched Activation Z-Scores when canonical pathways share their directional gene set), and ribosomal quality control signaling showed a z-score of 5.385 with a −log(*p* value) of 19.7. Genes contributing to these enriched pathways included ribosomal and translational quality-control components such as Rpl11, Rpl13, Rps27a, Psmb4, and Uba52. In addition, increased expression of Gpx4 within these pathway analyses was observed in the FOD-CCI group relative to SOD-CCI.

**Figure 3:**
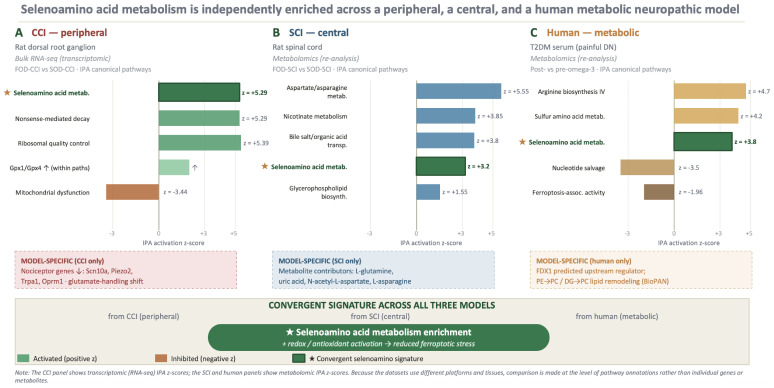
Cross-model convergence of omega-3-responsive pathways identifies selenoamino acid metabolism as a shared signature across peripheral, central, and human metabolic neuropathic models. IPA activation z-scores for omega-3-associated canonical pathways are shown side by side for three independent datasets: (**A**) rat DRG bulk RNA-sequencing data from the FOD-CCI versus SOD-CCI comparison (peripheral nerve injury); (**B**) rat spinal cord metabolomic data from the FOD-SCI versus SOD-SCI comparison (central nervous system injury); and (**C**) human serum metabolomic data from omega-3-supplemented participants with type 2 diabetes (metabolic neuropathy). In each panel, horizontal bars extend rightward for pathways with positive activation z-scores (predicted activation) and leftward for negative z-scores (predicted inhibition), with values shown adjacent to each bar. Bar color denotes the model (coral, CCI; blue, SCI; amber, human), and selenoamino acid metabolism is shown in green and marked with a star (★) in all three panels to highlight the signature shared across models. Color shading is used only to distinguish the three models and does not encode additional quantitative information. The convergence of selenoamino acid metabolism across all three models, despite their distinct etiologies, tissues, and analytical platforms, identifies it as the principal convergent omega-3-responsive signature. Because the CCI dataset is transcriptomic and the SCI and human datasets are metabolomic, comparison across models is made at the level of pathway annotations rather than individual genes or metabolites.

We then examined whether similar pathway-level changes were present in a central nervous system injury model by re-analyzing previously generated spinal cord metabolomic data from the FOD-SCI versus SOD-SCI comparison using IPA (Figure 2E). This analysis identified activation of transport of bile salts and organic acids and aspartate and asparagine metabolism, with metabolite contributors including L-glutamine, uric acid, N-acetyl-L-aspartic acid, and L-asparagine. In addition, nicotinate metabolism and glycerophospholipid biosynthesis were activated. Notably, selenoamino acid metabolism was also enriched in the SCI dataset (z = 3.2), matching the pattern observed in the DRG transcriptomic analysis of the CCI model. This cross-model convergence of selenoamino acid metabolism across the peripheral (CCI), central (SCI), and human metabolic datasets is summarized in Figure 3, which presents the IPA pathway-enrichment z-scores for all three models side by side.

Taken together, these data show that omega-3 supplementation was associated with a smaller injury-induced transcriptional shift in DRG, reduced representation of inflammatory and mitochondrial dysfunction programs, and enrichment of pathways linked to selenoamino acid metabolism, mRNA surveillance, and translational quality control.

### 3.3. Omega-3 Supplementation Alters Expression of Pain-Associated Genes in DRG Following CCI

**Figure 4:**
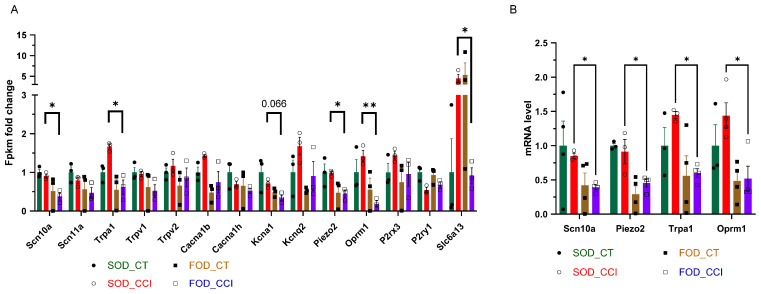
Omega-3 supplementation alters expression of pain-associated genes in DRG after CCI. Pain-associated gene expression was evaluated in DRG tissue 7 days after CCI. (**A**) RNA-seq analysis of FOD-CCI versus SOD-CCI animals showing differential expression of genes associated with nociceptive signaling, ion channel activity, mechanosensation, purinergic signaling, and glutamate-related pathways. Genes shown include voltage-gated sodium channels, transient receptor potential channels, calcium and potassium channel genes, mechanosensitive channels, purinergic receptors, glutamate-associated genes, and opioid receptor-associated genes. (**B**) Quantitative RT-PCR validation of selected pain-associated genes, including Scn10a, Piezo2, Trpa1, and Oprm1. Expression values are normalized to β-actin and expressed relative to control samples. Data are shown as mean ± SEM; *n* = 4 rats per group unless otherwise indicated. Statistical analysis was performed using ANOVA with Tukey’s post hoc test or as indicated in Section 2. Statistical significance is indicated in each panel. * *p* < 0.05, ** *p* < 0.01.

Because the transcriptomic analysis identified broad omega-3-associated changes in DRG pathways after CCI, we next examined genes directly linked to nociceptive signaling, ion channel activity, and pain-associated neuronal excitability. FPKM fold-change values were evaluated for selected pain-related genes in the FOD-CCI versus SOD-CCI comparison at 1 week post-injury (Figure 4A).

Several voltage-gated sodium channel genes and sensory transduction genes were reduced in the FOD-CCI group relative to the SOD-CCI group. Scn10a, which encodes Nav1.8 and is strongly associated with nociceptor excitability, was decreased in FOD-CCI animals (log2FC = −1.26, adjusted *p* < 0.05). Piezo2, a mechanosensitive ion channel involved in touch and mechanical sensitivity, was also reduced in FOD-CCI animals compared with SOD-CCI animals (log2FC = −0.78, adjusted *p* < 0.01). Trpa1, a nociceptive transient receptor potential channel associated with chemical and oxidative stress sensitivity, was decreased in the FOD-CCI group (log2FC = −1.36, adjusted *p* < 0.05).

Additional pain-associated genes followed a similar directional pattern. Expression of Oprm1, encoding the μ-opioid receptor, was reduced in FOD-CCI animals relative to SOD-CCI animals (log2FC = −2.8, adjusted *p* < 0.01). Other genes involved in nociceptive signaling, including voltage-gated sodium channels, calcium channels, potassium channels, purinergic receptors, and glutamatergic signaling components, also showed differential expression patterns between diet groups (Figure 4A).

To validate selected RNA-seq findings, quantitative RT-PCR was performed for Scn10a, Piezo2, Trpa1, and Oprm1 (Figure 4B). RT-PCR analysis confirmed the directionality of expression changes observed in the RNA-seq dataset, with reduced expression of these pain-associated genes in FOD-CCI animals relative to SOD-CCI animals.

Together, these data show that omega-3 supplementation was associated with reduced expression of multiple genes involved in nociceptive excitability and pain signaling in DRG tissue after CCI.

### 3.4. Omega-3 Supplementation Increases Antioxidant-Associated Gene and Protein Markers in DRG

Following pathway enrichment showing activation of selenoamino acid metabolism in the FOD-CCI versus SOD-CCI comparison, we next examined individual genes associated with antioxidant defense, selenium metabolism, and lipid peroxide detoxification.

**Figure 5:**
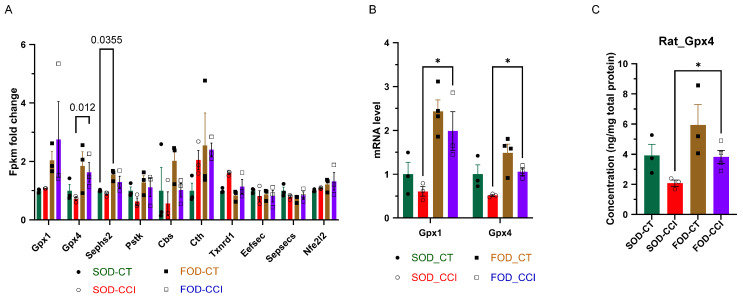
Omega-3 supplementation increases antioxidant-associated markers and GPX4 protein in DRG. Antioxidant and selenoamino acid metabolism-associated markers were evaluated in DRG tissue 7 days after CCI. (**A**) RNA-seq analysis showing increased expression of antioxidant- and selenium-associated genes in FOD-CCI animals relative to SOD-CCI animals, including Gpx1, Gpx4, Sephs2, Pstk, and related pathway genes. (**B**) Quantitative RT-PCR validation of Gpx1 and Gpx4 expression. (**C**) ELISA quantification of GPX4 protein levels in DRG tissue. Increased GPX4 mRNA and protein levels are consistent with enhanced antioxidant capacity and reduced susceptibility to ferroptosis-associated oxidative stress. Data are shown as mean ± SEM; *n* = 4 rats per group for gene-expression analyses and *n* as indicated for ELISA. Statistical analysis was performed using ANOVA with Tukey’s post hoc test or as indicated in Section 2. Statistical significance is indicated in each panel. * *p* < 0.05.

FPKM analysis demonstrated increased expression of several antioxidant- and selenoprotein-related genes in the FOD-CCI group relative to the SOD-CCI group (Figure 5A). Gpx1 showed an average fold increase of 1.41 in FOD-CCI animals compared with SOD-CCI animals, while Gpx4 showed an average fold increase of 1.60. Additional genes involved in selenium-related metabolism and antioxidant pathway regulation also increased, including Sephs2 (fold change = 1.50), Pstk (fold change = 1.72), and Cbs (fold change = 1.66).

To validate selected antioxidant-related transcripts, quantitative RT-PCR was performed for Gpx1 and Gpx4. RT-PCR confirmed increased expression of both genes in FOD-CCI animals relative to SOD-CCI controls. Gpx1 increased by 1.60-fold (*p* < 0.01), and Gpx4 increased by 1.45-fold (*p* < 0.05) (Figure 5B).

Because GPX4 is a central antioxidant enzyme involved in reducing lipid hydroperoxides, GPX4 protein levels were further assessed by ELISA in DRG tissue. FOD-CCI animals exhibited increased GPX4 protein levels relative to SOD-CCI animals, with an average fold increase of 1.50 (*p* < 0.05) (Figure 5C).

These transcript- and protein-level data demonstrate that omega-3 supplementation increased antioxidant-associated markers in DRG tissue after CCI, including GPX4 at both mRNA and protein levels.

### 3.5. Omega-3 Supplementation Modulates EIF2AK4-Associated Stress Response and Translational Quality-Control Genes

Because pathway analysis identified enrichment of NMD, ribosomal quality control, and EIF2AK4-associated stress response pathways, we next evaluated genes contributing to these processes in the FOD-CCI versus SOD-CCI comparison.

**Figure 6:**
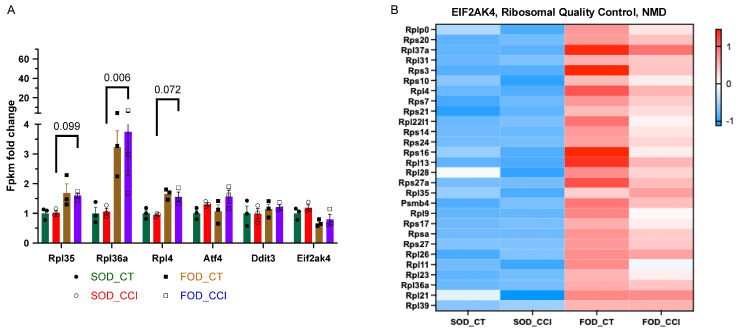
Omega-3 supplementation modulates EIF2AK4-associated stress-response and translational quality-control pathways in DRG. Genes associated with EIF2AK4 signaling, ribosomal quality control, NMD, and translational stress responses were evaluated in DRG tissue after CCI. (**A**) RNA-seq fold-change analysis of selected EIF2AK4-associated and translational stress-response genes, including Rpl35, Rpl36a, Rpl4, Atf4, Ddit3, and Eif2ak4, comparing FOD-CCI and SOD-CCI animals. (**B**) Heatmap showing coordinated expression differences among ribosomal and protein quality-control genes, including Rplp0, Rps20, Rpl37a, Rpl31, Rps3, Rps10, Rpl4, Rps7, Rps21, Rpl22l1, Rps14, Rps24, Rps16, Rpl28, Rps27a, Rpl35, Psmb4, Rpl9, Rps17, Rpsa, Rps27, Rpl26, Rpl11, Rpl23, and Rpl39. Data are shown as mean ± SEM where applicable; *n* = 4 rats per group. Statistical analysis was performed as described in Section 2.

FPKM fold-change analysis showed increased expression of several ribosomal and translational stress response genes in FOD-CCI animals relative to SOD-CCI animals (Figure 6A). Rpl35 increased by 1.58-fold, Rpl36a increased by 3.42-fold, and Rpl4 increased by 1.63-fold in FOD-CCI animals compared with SOD-CCI animals. Stress response-associated genes also showed increased expression, including Atf4 (fold change = 1.31) and Ddit3 (fold change = 1.33). In contrast, Eif2ak4 showed a relative decrease in FOD-CCI animals compared with SOD-CCI animals (fold change = −1.32).

A heatmap of additional genes associated with ribosomal function, protein synthesis, and quality-control pathways showed coordinated expression differences between FOD-CCI and SOD-CCI groups (Figure 6B). Genes represented in this cluster included Rplp0, Rps20, Rpl37a, Rpl31, Rps3, Rps10, Rpl4, Rps7, Rps21, Rpl22l1, Rps14, Rps24, Rps16, Rpl28, Rps27a, Rpl35, Psmb4, Rpl9, Rps17, Rpsa, Rps27, Rpl26, Rpl11, Rpl23, and Rpl39.

These data indicate that omega-3 supplementation was associated with altered expression of genes involved in translational regulation, ribosomal quality control, and cellular stress response pathways in DRG tissue after CCI.

### 3.6. Human Serum Metabolomic Re-Analysis Identifies Omega-3-Associated Enrichment of Selenoamino Acid and Redox-Related Pathways

To assess whether pathways identified in the rat CCI and SCI models were also represented in human diabetic neuropathy-related datasets, previously generated serum metabolomic data from omega-3-supplemented participants with type 2 diabetes were re-analyzed using IPA software. This analysis was used to identify canonical pathways and predicted upstream regulators associated with omega-3 supplementation in the human cohort.

**Figure 7:**
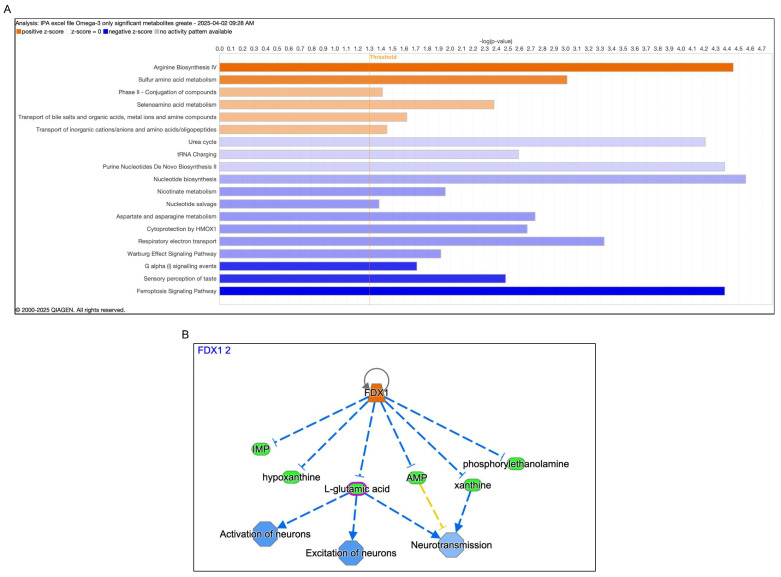
Human serum metabolomic re-analysis identifies omega-3-associated enrichment of selenoamino acid metabolism and predicted FDX1 upstream regulation. Previously generated serum metabolomic data from omega-3–supplemented participants with type 2 diabetes were re-analyzed using IPA. (**A**) Canonical pathway analysis showing enrichment of pathways including selenoamino acid metabolism, arginine biosynthesis, sulfur amino acid metabolism, nucleotide salvage, tRNA charging, and predicted ferroptosis-associated pathway activity. Bar length represents statistical significance [−log(p-value)], and the vertical line indicates the significance threshold. Bar color indicates the predicted activation state based on the IPA z-score: orange denotes positive z-scores (predicted activation), blue denotes negative z-scores (predicted inhibition), grey denotes a z-score of zero, and pale bars indicate pathways with no activity pattern available. (**B**) Upstream regulator analysis identifying FDX1 as a predicted upstream regulator associated with omega-3–related metabolomic changes. Node color and shape follow standard IPA conventions: FDX1 (the upstream regulator) is shown in orange, metabolites in green, and predicted downstream functions in blue; connecting edges indicate predicted relationships (for example, activation or inhibition) as defined by IPA. FDX1 was identified computationally and was not functionally validated in this study. IPA disease/function annotation outputs supporting the predicted reduction in ferroptosis-associated pathway activity are provided in Supplementary Table S5.

IPA analysis of serum metabolomic changes following omega-3 supplementation identified several enriched canonical pathways (Figure 7A). Activated pathways included arginine biosynthesis IV (z-score = 4.7; −log(*p* value) = 4.72), sulfur amino acid metabolism (z-score = 4.2; −log(*p* value) = 4.15), and selenoamino acid metabolism (z-score = 3.8; −log(*p* value) = 3.67). Inhibited pathways included nucleotide salvage (z-score = −3.5; −log(*p* value) = 3.45) and RNA charging (z-score = −3.0; −log(*p* value) = 3.12). The enrichment of selenoamino acid metabolism in the human serum dataset overlapped with pathway findings from the rat CCI DRG transcriptomic analysis and the rat SCI metabolomic re-analysis.

Disease and function annotation analyses further identified predicted reductions in several cell death-associated pathways following omega-3 supplementation. In the human serum metabolomic dataset, predicted annotations included reduced cell death of tumor cell lines (*p* = 0.00245; activation z-score = −2.459; 12 molecules), reduced ferroptosis-associated pathway activity (*p* = 0.00251; activation z-score = −1.957; 4 molecules), reduced necrosis (*p* = 0.014; activation z-score = −1.839; 15 molecules), reduced cell death of immune cells (*p* = 0.00647; activation z-score = −1.638; 7 molecules), and reduced apoptosis of tumor cell lines (*p* = 0.0311; activation z-score = −1.564; 8 molecules). In contrast, annotations related to cell viability were predicted to increase, including cell viability of tumor cell lines (*p* = 0.00154; activation z-score = 1.644; 9 molecules) and cell viability (*p* = 0.00264; activation z-score = 1.993; 11 molecules). These disease/function annotation outputs are provided in Supplementary Table S5.

These disease/function annotations were derived from IPA-based computational predictions and therefore represent pathway-level associations rather than direct measurements of cell death. Accordingly, the ferroptosis annotation was interpreted as a predicted reduction in ferroptosis-associated pathway activity.

Upstream regulator analysis identified FDX1 as a predicted upstream regulator associated with the metabolomic changes observed after omega-3 supplementation (z-score = 2.42; *p* = 4.13 × 10^−5^) (Figure 7B). FDX1 was retained as a computationally predicted regulatory node for pathway-level interpretation and was not used as direct evidence of functional causality.

Together, these findings show that omega-3 supplementation in the human diabetic cohort was associated with enrichment of amino acid metabolism, selenoamino acid metabolism, and redox-related pathways, along with predicted reductions in ferroptosis-associated pathway activity. These human serum findings overlap with pathway patterns observed in the rat CCI and SCI analyses. The human IPA and FDX1 upstream regulator results are shown in Figure 7A,B, and the corresponding IPA disease/function annotation outputs are provided in Supplementary Table S5.

### 3.7. BioPAN Lipidomic Analysis Reveals Omega-3-Associated Remodeling of Human Serum Lipid Pathways

To further evaluate lipid remodeling after omega-3 supplementation, human serum lipidomic data were re-analyzed using BioPAN. This analysis focused on lipid class and lipid species pathway transitions involving monoacylglycerol (MG), diacylglycerol (DG), phosphatidylcholine (PC), and phosphatidylethanolamine (PE) lipid pathways.

**Figure 8:**
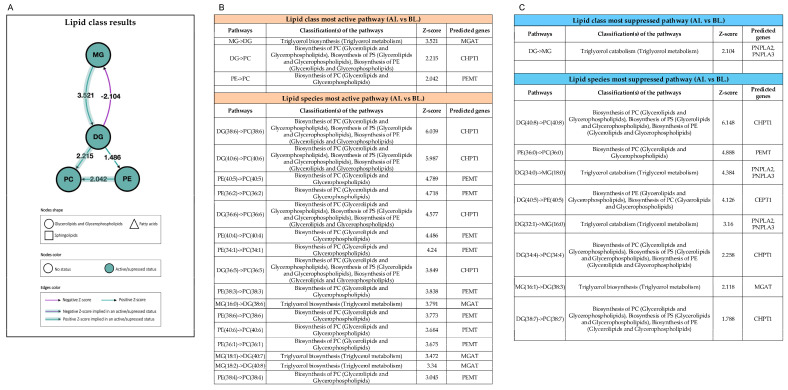
BioPAN lipidomic analysis identifies omega-3-associated remodeling of MG, DG, PC, and PE lipid pathways in human serum. Human serum lipidomic data from omega-3-supplemented participants with type 2 diabetes were re-analyzed using BioPAN. (**A**) The pathway network shows omega-3-associated lipid class and lipid species transitions involving MG, DG, PC, and PE. BioPAN identified activated MG-to-DG, DG-to-PC, and PE-to-PC pathway transitions, as well as suppressed DG-to-MG and selected species-level transitions. Detailed active lipid class and species transitions are provided in panel (**B**), and suppressed lipid class and species transitions are provided in panel (**C**).

At the lipid class level, BioPAN identified activation of the MG-to-DG transition following omega-3 supplementation (z-score = 3.521; predicted enzyme MGAT), together with activation of the downstream DG-to-PC transition (z-score = 2.215). These class-level transitions, together with the corresponding suppressed transitions described below, are summarized in the BioPAN pathway network (Figure 8A), which provides an integrated overview of the detailed activated and suppressed transitions reported in Figure 8B and Figure 8C, respectively.

Resolving these changes to the level of individual lipid species, BioPAN identified the specific molecular transitions driving the activated pathways (Figure 8B). The most strongly activated species-level transitions were DG(38:6)-to-PC(38:6) (z-score = 6.039; CHPT1), DG(40:6)-to-PC(40:6) (z-score = 5.987; CHPT1), PE(40:5)-to-PC(40:5) (z-score = 4.789; PEMT), PE(36:2)-to-PC(36:2) (z-score = 4.718; PEMT), and DG(36:6)-to-PC(36:6) (z-score = 4.577; CHPT1). Additional activated PE-to-PC transitions included PE(40:4)-to-PC(40:4), PE(34:1)-to-PC(34:1), PE(38:3)-to-PC(38:3), PE(38:6)-to-PC(38:6), PE(40:6)-to-PC(40:6), PE(36:1)-to-PC(36:1), and PE(38:4)-to-PC(38:4), and activated MG-to-DG species transitions included MG(16:0)-to-DG(38:6), MG(18:1)-to-DG(40:7), and MG(18:2)-to-DG(40:8). The predominance of PE-to-PC and DG-to-PC transitions among the most strongly activated species indicates that omega-3 supplementation preferentially routed polyunsaturated acyl chains toward PC.

BioPAN also identified suppressed lipid pathway transitions following omega-3 supplementation. At the lipid class level, the DG-to-MG pathway was predicted to be reduced (z-score = −2.104), with PNPLA2 and PNPLA3 identified as predicted enzymes (Figure 8C). At the species level, suppressed transitions included DG(40:8)-to-PC(40:8) (z-score = −6.148; predicted enzyme CHPT1), PE(36:0)-to-PC(36:0) (z-score = −4.888; predicted enzyme PEMT), DG(34:0)-to-MG(18:0) (z-score = −4.384; predicted enzymes PNPLA2 and PNPLA3), and DG(40:5)-to-PE(40:5) (z-score = −4.126; predicted enzyme CEPT1) (Figure 8C). Additional suppressed species-level transitions included DG(32:1)-to-MG(16:0), DG(34:4)-to-PC(34:4), MG(16:1)-to-DG(38:3), and DG(38:7)-to-PC(38:7).

Together, these data show that omega-3 supplementation was associated with coordinated remodeling of human serum lipid pathways involving MG, DG, PC, and PE lipid classes. The presence of both activated and suppressed lipid species transitions indicates that omega-3 supplementation did not uniformly increase or decrease lipid remodeling, but instead altered specific lipid class and species-level pathways.

### 3.8. Rat DRG Glutamate and Lipid Metabolism Gene Patterns Align with Human Metabolomic and Lipidomic Pathway Changes

To determine whether human serum pathway-level findings aligned with tissue-level gene-expression changes in the CCI model, rat DRG RNA-seq data were examined for genes involved in glutamate handling and lipid metabolism. These analyses focused on the FOD-CCI versus SOD-CCI comparison and were used to evaluate whether omega-3 supplementation altered pathways related to excitatory neurotransmitter metabolism and lipid remodeling in injured DRG tissue.

**Figure 9:**
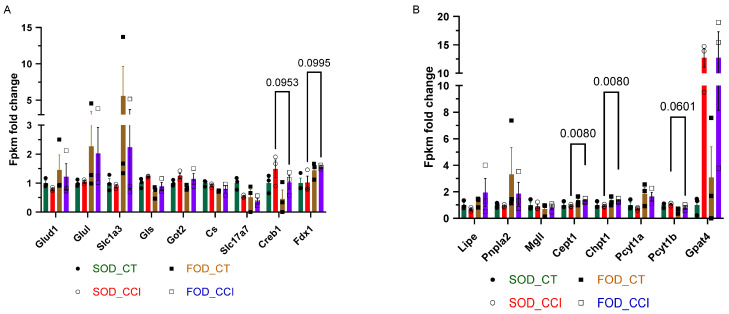
Omega-3 supplementation alters glutamate-handling and lipid-metabolism gene expression in rat DRG after CCI. Rat DRG RNA-seq data were analyzed to evaluate genes involved in glutamate handling and lipid metabolism in FOD-CCI versus SOD-CCI animals. (**A**) Expression patterns of glutamate-handling genes, including Glul, Slc1a3, Gls, Slc17a7, Glud1, Creb1, Cs, and Fdx1. (**B**) Expression patterns of lipid-metabolism genes, including Lipe, Pnpla2, Mgll, Cept1, Chpt1, Pcyt1a, Pcyt1b, and Gpat4. Data are shown as mean ± SEM where applicable; *n* = 4 rats per group. Statistical analysis was performed as described in Section 2.

Glutamate-handling transcripts showed diet-associated differences in FOD-CCI animals relative to SOD-CCI animals (Figure 9A). Glul, which encodes glutamine synthetase and participates in glutamate-to-glutamine conversion, was increased in FOD-CCI animals relative to SOD-CCI animals. Slc1a3, which encodes the excitatory amino acid transporter EAAT1 and contributes to glutamate uptake, was also increased in the FOD-CCI group. In contrast, Gls, which encodes glutaminase and contributes to glutamate production, was reduced in FOD-CCI animals relative to SOD-CCI animals. Slc17a7, which encodes the vesicular glutamate transporter VGLUT1, was also reduced in the FOD-CCI group. Glud1 showed an upward trend in FOD-CCI animals, while Creb1 and Cs showed relative reductions compared with SOD-CCI animals. Fdx1 was increased in FOD-CCI animals relative to SOD-CCI animals, consistent with its identification as a predicted upstream regulator in the human serum metabolomic analysis.

A distinct set of lipid-metabolism transcripts also differed between the FOD-CCI and SOD-CCI groups (Figure 9B). Lipe, which is involved in lipid mobilization, was increased in FOD-CCI animals relative to SOD-CCI animals. Pnpla2, which contributes to triglyceride hydrolysis, was also increased in the FOD-CCI group. Mgll, which participates in MG metabolism, remained relatively stable across groups. Transcripts involved in phospholipid synthesis and remodeling, including Cept1, Chpt1, and Pcyt1a, were increased in FOD-CCI animals relative to SOD-CCI animals. Pcyt1b was reduced in FOD-CCI animals, while Gpat4, which participates in glycerolipid and phospholipid biosynthesis, was increased in CCI groups relative to contralateral controls.

Disease/function annotation analysis of the rat spinal cord metabolomic dataset further identified pathway-level changes in cell death- and survival-associated categories following omega-3 supplementation. In the FOD-SCI versus SOD-SCI comparison, IPA predicted reduced activity in several cell death-associated annotations, including central nervous system cell death, cerebral cortex cell death, neuronal cell death, and ferroptosis-associated pathway activity. IPA also predicted increased activity in selected cell viability and survival-associated annotations. These rat spinal cord metabolomic disease/function outputs are provided in Supplementary Table S6.

Together, these DRG gene-expression and spinal cord metabolomic data show that omega-3 supplementation was associated with changes in glutamate-handling, lipid-metabolism, and cell stress-associated pathways across injury models. The DRG glutamate and lipid gene-expression patterns shown in Figure 9A,B correspond to pathway categories identified in the human serum metabolomic and lipidomic analyses, including amino acid/redox metabolism and MG, DG, PC, and PE lipid pathway remodeling.

## 4. Discussion

The present study supports the concept that ferroptotic stress, rather than necessarily overt ferroptotic cell death, may represent a convergent biological feature [27,28] across multiple NP contexts, including CCI, SCI, and pDN. Using behavioral, transcriptomic, metabolomic, and lipidomic analyses, we found that omega-3 PUFA supplementation was associated with reduced NP-related behaviors in the CCI model and coordinated modulation of pathways linked to redox regulation, selenoamino acid metabolism, lipid remodeling, glutamate handling, and cellular stress responses. We interpret these data as evidence of pathway-level ferroptotic stress and antioxidant adaptation, not as direct demonstration of ferroptotic cell death. This distinction is critical because NP may arise from persistent neuronal dysfunction driven by lipid peroxidation burden, mitochondrial stress, altered membrane composition, and impaired antioxidant buffering before irreversible cell death occurs.

The behavioral findings provide the functional foundation for this mechanistic analysis. In the CCI model, animals receiving the FOD showed attenuation of thermal hypersensitivity and improvement in multiple CatWalk gait parameters compared with SOD-fed injured animals (Figure 1). These behavioral outcomes were observed across sensory and locomotor domains, indicating that omega-3 supplementation affected both pain-related sensitivity and functional recovery after peripheral nerve injury. The absence of comparable behavioral changes in sham animals supports the interpretation that the observed effects were most evident under injury-induced neuropathic stress rather than reflecting nonspecific changes in baseline locomotion.

Transcriptomic profiling of DRG tissue provided the first molecular layer connecting omega-3 supplementation to injury-associated pathway modulation. SOD-fed CCI animals showed a broader injury-induced transcriptional response, including inflammatory and injury-responsive gene expression changes, whereas omega-3-treated CCI animals showed a more restricted transcriptomic shift. Pathway analysis identified reduced representation of mitochondrial dysfunction and enrichment of selenoamino acid metabolism, NMD, and ribosomal quality-control pathways in the omega-3 group (Figure 2). This pattern is important because mitochondrial dysfunction, impaired protein quality control, and oxidative stress can increase cellular susceptibility to lipid peroxide accumulation [27,29]. Rather than indicating a single isolated mechanism, the DRG transcriptomic data suggest that omega-3 supplementation shifts the injured sensory ganglion toward a state with greater antioxidant and stress-adaptive capacity.

The most consistent mechanistic signal across datasets was the activation of selenoamino acid metabolism. In the DRG, omega-3 supplementation increased the expression of antioxidant and selenium-associated genes, including Gpx1, Gpx4, Sephs2, Pstk, and Cbs. RT-PCR confirmed increased Gpx1 and Gpx4 expression, and ELISA confirmed increased GPX4 protein levels in DRG tissue (Figure 5). This is central to the ferroptotic stress model because GPX4 is a major enzymatic defense against lipid hydroperoxide accumulation [27,28,30]. However, increased GPX4 should not be interpreted as proof that ferroptotic cell death occurred or was blocked. Instead, it indicates enhanced antioxidant capacity within a pathway biologically positioned to buffer lipid peroxidation and ferroptosis-associated stress [31]. An alternative interpretation is that GPX4 induction reflects a generic antioxidant response rather than a specifically ferroptosis-relevant adaptation, since GPX4 levels rise in many oxidative stress contexts. We consider the broader pathway pattern observed here—coordinated activation of selenoamino acid metabolism, induction of Sephs2, Pstk, and Cbs, and reduced mitochondrial dysfunction signature—to collectively distinguish the omega-3 response from a generic antioxidant induction and to point specifically toward a selenium-dependent ferroptotic-stress defense program.

This distinction between ferroptotic stress and ferroptotic cell death is essential for interpreting NP biology. In chronic pain states, sensory neurons and spinal cord circuits may remain viable but functionally altered by persistent oxidative and lipid metabolic stress. Sublethal ferroptotic stress could contribute to pain by altering membrane excitability, mitochondrial function, lipid signaling, and neuron–glia communication without producing immediate cell loss [9,10]. Therefore, the relevance of ferroptosis biology in this manuscript is not limited to cell death [32,33,34]. This conceptualization builds directly on the framework developed by Maher and colleagues, who proposed ferroptosis-related stress as a unifying biological state in which the upstream lipid-peroxidation, iron-handling, and selenium-dependent antioxidant programs that culminate in ferroptotic death are themselves drivers of cellular dysfunction in non-lethal contexts [35]. This concept has recently been extended to the central and peripheral nervous systems, with ferroptosis-related stress signatures demonstrated in Niemann–Pick disease type C, a lysosomal-storage disease with prominent CNS pathology [33], and in Charcot–Marie–Tooth disease type 1A, the most common inherited peripheral neuropathy [34]. The present findings extend this concept from inherited neurodegenerative disease to acquired NP, supporting ferroptotic stress as a shared biological state across CNS and PNS injury and disease contexts [14,16]. Rather, the data support a model in which neuropathic injury and metabolic disease increase ferroptosis-associated stress burden, while omega-3 supplementation enhances protective systems that reduce this burden.

The enrichment of EIF2AK4-associated stress response, NMD, and ribosomal quality-control pathways adds another layer to the protective profile observed in omega-3-treated animals. Neuropathic injury imposes substantial stress on sensory neurons, including mitochondrial dysfunction, oxidative stress, altered translation, and protein misfolding [35,36]. Increased expression of ribosomal and translational quality-control genes in the FOD-CCI group indicates that omega-3 supplementation was associated with changes in pathways involved in maintaining protein homeostasis during injury (Figure 6). These findings integrate with the selenoamino acid and GPX4 data by suggesting that omega-3 supplementation acts through a broader stress-adaptive program involving redox control, protein quality control, and metabolic remodeling. Mechanistically, the EIF2AK4/ATF4 axis is not parallel to but converges with ferroptosis biology: ATF4 transcriptionally regulates components of system xc^−^ (Slc7a11) and other glutathione-pathway genes that modulate ferroptotic susceptibility [35,36]. The coordinated enrichment of integrated stress response and selenoamino acid pathways observed here is therefore consistent with a single, integrated redox–protein–quality-control program, rather than two unrelated signatures.

Omega-3 supplementation also altered genes associated with nociceptive signaling in the DRG. Reduced expression of Scn10a, Piezo2, Trpa1, and Oprm1 [37,38,39] was observed in omega-3-treated CCI animals relative to control CCI animals and confirmed for selected targets by RT-PCR (Figure 4). These genes are involved in nociceptor excitability, mechanical sensitivity, oxidative stress sensing, and opioid receptor signaling. In the context of the behavioral data, reduced expression of these pain-associated genes provides a molecular correlate of decreased thermal hypersensitivity and improved gait outcomes. Of these targets, Oprm1 warrants interpretive caution: reduced μ-opioid receptor expression could reflect compensatory down-regulation in response to reduced ongoing nociceptive drive in the omega-3 group, a finding consistent with the behavioral data, or alternatively could signal a reduction in endogenous analgesic capacity, which would not be desirable. The Scn10a, Piezo2, and Trpa1 reductions are more straightforwardly aligned with reduced nociceptive excitability; functional electrophysiological validation will be necessary to confirm that transcript-level changes translate to altered channel function. More broadly, these changes may reflect downstream consequences of altered redox and lipid stress signaling, although the direction of causation between selenium-dependent antioxidant induction, lipid remodeling, and pain-gene expression remains to be tested directly.

Glutamate regulation emerged as another point of convergence in the rat DRG dataset. Omega-3 supplementation was associated with increased expression of Glul and Slc1a3 and reduced expression of Gls and Slc17a7 in FOD-CCI animals relative to SOD-CCI animals (Figure 9A). This pattern indicates altered expression of genes involved in glutamate uptake, glutamine cycling, and excitatory neurotransmitter handling after injury. Because glutamate dysregulation contributes to neuronal hyperexcitability and sensitization [7], these findings provide a complementary molecular link between omega-3 supplementation and reduced NP-associated behavioral changes. These glutamate-related changes also fit within the broader redox model, as mitochondrial dysfunction and oxidative stress can disrupt glutamate metabolism, while excessive glutamate signaling can further amplify oxidative stress [27].

The human re-analysis strengthens the translational relevance of the preclinical findings. In serum from omega-3-supplemented participants with type 2 diabetes, pathway analysis identified activation of arginine biosynthesis, sulfur amino acid metabolism, and selenoamino acid metabolism. The recurrence of selenoamino acid metabolism in both rat and human datasets is one of the strongest cross-model observations in the study. Human metabolomic analysis also predicted reduced ferroptosis-associated pathway activity and identified FDX1 as a predicted upstream regulator (Figure 7). Because FDX1 was identified computationally and was not experimentally manipulated, it should be viewed as a candidate regulatory node rather than a validated mediator. Notably, FDX1 sits at the intersection of mitochondrial iron-sulfur biology, protein lipoylation, and Cu/Fe redox cycling, and is now more strongly associated with cuproptosis than with ferroptosis per se. We therefore interpret its appearance as a marker of broader mitochondrial-redox dysregulation that is responsive to omega-3 supplementation, rather than as evidence of ferroptosis-specific regulation. Its appearance in the human pathway analysis, together with increased Fdx1 expression in rat DRG, nonetheless provides a rationale for future mechanistic studies testing whether FDX1 links omega-3 supplementation to mitochondrial redox regulation and selenium-dependent antioxidant responses [40,41].

The lipidomic findings provide a key link between omega-3 supplementation and ferroptotic stress vulnerability. Ferroptotic stress is driven by accumulation of lipid peroxides within susceptible membrane phospholipids [27,42]. In the human serum lipidomics re-analysis, BioPAN identified coordinated remodeling of MG, DG, PC, and PE lipid pathways after omega-3 supplementation, including increased activity of DG-to-PC and PE-to-PC biosynthetic routes (Figure 8). Species-level BioPAN analysis identified multiple activated PE-to-PC transitions enzymatically attributed to PEMT, including PE(40:5)-to-PC(40:5), PE(36:2)-to-PC(36:2), and additional PUFA-containing PE species, alongside activated DG-to-PC transitions attributed to CHPT1. This pattern offers a candidate mechanistic resolution to the apparent paradox of how a PUFA-class intervention reduces, rather than amplifies, ferroptotic stress. PE species, particularly those esterified to arachidonic and adrenic acid (PE-AA and PE-AdA), are the canonical substrates of ferroptotic lipid peroxidation [43], whereas the equivalent PUFA-containing species esterified into PC are markedly less susceptible to peroxidation-driven membrane damage. The omega-3-associated activation of PE-to-PC conversion observed here would therefore be expected to redistribute long-chain PUFAs from a peroxidation-prone PE pool into a comparatively peroxidation-resistant PC pool, while the parallel activation of DG-to-PC biosynthesis (CHPT1) and reduction in DG-to-PE channeling reinforce this directional shift at the glycerolipid precursor level [43]. In this model, dietary omega-3 PUFAs are not simply added to the membrane phospholipid pool; rather, they are accompanied by remodeling enzymes that preferentially route them into membrane compartments less vulnerable to ferroptotic peroxidation. This biochemical logic provides a plausible explanation for how a PUFA-class intervention can reduce, rather than amplify, ferroptotic stress, and it nominates PEMT- and CHPT1-mediated phospholipid remodeling as specific points of convergence between omega-3 supplementation and ferroptosis biology. These lipid pathway changes indicate that omega-3 supplementation altered lipid class transitions and phospholipid remodeling, rather than simply increasing omega-3 fatty acid abundance [43]. In rat DRG, expression changes in lipid metabolism genes, including Cept1, Chpt1, Pcyt1a, Lipe, and Pnpla2, aligned with the human lipidomic pathway findings (Figure 9B). Together, these data support a model in which omega-3 supplementation modifies the lipid environment in a way that may reduce vulnerability to oxidative lipid stress. Direct measurement of oxidized phospholipid species in DRG or spinal cord tissue will be needed to confirm this mechanism.

Across the three models examined, CCI, SCI, and pDN, the shared signal was not a single gene, metabolite, or pathway, but a recurring biological pattern: omega-3 supplementation was associated with enhanced antioxidant capacity, modulation of selenium-dependent metabolism, altered lipid remodeling, and reduced pathway-level signatures consistent with ferroptosis-associated oxidative stress. CCI represents peripheral nerve injury, SCI represents central neurotrauma, and pDN represents metabolic neuropathy. Despite these distinct initiating insults, all three conditions involve oxidative stress, mitochondrial dysfunction, lipid dysregulation, and neuronal stress [7,28]. Our integrated analysis suggests that ferroptotic stress may represent one shared downstream vulnerability across NP states. Because the CCI dataset is transcriptomic and the SCI and human datasets are metabolomic, the models cannot be compared at the level of individual genes or metabolites; the comparison is therefore made at the level of pathway annotations, as summarized across the three models in Figure 3.

Several limitations should be acknowledged. First, this study does not directly demonstrate ferroptotic cell death. The data support pathway-level ferroptotic stress, but direct assays of lipid peroxidation, iron handling, oxidized phospholipid species, mitochondrial morphology, and rescue with ferroptosis-pathway modulators would be required to establish causality. Second, the DRG RNA-seq sample size was modest, and the adjusted *p*-value threshold of ≤0.1 was used to identify pathway-level signals for validation rather than definitive gene-level discovery. Key findings were supported by RT-PCR and ELISA, but additional validation in larger cohorts is warranted. Third, gene-expression changes in glutamate-handling and lipid-metabolism pathways require validation at the protein and cell-specific levels using approaches such as immunohistochemistry, spatial transcriptomics, or single-cell RNA sequencing. Fourth, the human analysis was a secondary analysis of a previously published single-arm intervention, limiting causal interpretation and generalizability. Fifth, FDX1 was identified as a predicted regulator and should be treated as a hypothesis-generating finding until functional studies test its role directly. Additional limitations include the use of only female rats, selected in part to maintain consistency with the SCI datasets re-analyzed here. Because NP mechanisms exhibit well-documented sex differences, the findings should be confirmed in male animals before generalization across sexes. Finally, because omega-3 PUFAs were delivered orally, we cannot distinguish effects mediated directly by parent fatty acids from those mediated by host-derived lipid mediators or microbiome-associated metabolism. However, this does not diminish the biological relevance of the findings, since microbiome interaction is an inherent component of dietary omega-3 supplementation, and the recurrence of redox, selenoamino acid, and lipid-remodeling pathway signatures across rat injury models and human diabetic datasets supports convergence on a shared downstream host-response program. Future studies incorporating microbiome profiling or targeted metabolite analysis could help define the relative contribution of microbiome-derived versus host-derived mediators.

Future studies should prioritize direct testing of the ferroptotic stress hypothesis across NP models. This includes quantifying oxidized PE and PC species, measuring 4-HNE or MDA, assessing iron-handling proteins, and determining whether ferroptosis-pathway modulators alter pain behaviors in CCI, SCI, and diabetic neuropathy models. Cell-specific studies are also needed to determine whether ferroptotic stress occurs primarily in sensory neurons, spinal neurons, Schwann cells, or infiltrating immune cells. Finally, prospective human studies with placebo-controlled omega-3 supplementation, paired pain phenotyping, and targeted lipid peroxidation biomarkers will be necessary to determine whether ferroptosis-associated oxidative stress predicts treatment response in pDN. Candidate response biomarkers for such trials include serum-oxidized PE and PC species, F2-isoprostanes, plasma selenium and selenoprotein P, and circulating GPX4 activity, any of which could enable biomarker-guided stratification of patients most likely to benefit from omega-3 intervention.

## 5. Conclusions

This study supports a model in which omega-3 supplementation attenuates NP-associated behavioral changes and modulates convergent redox and lipid metabolic pathways across the CCI, SCI, and pDN datasets. Across these models, omega-3 supplementation was associated with activation of selenoamino acid metabolism, increased GPX1/GPX4 expression, altered glutamate-handling pathways, and lipid remodeling consistent with reduced susceptibility to lipid peroxidation.

The central implication is not that ferroptotic cell death was directly demonstrated, but that ferroptotic stress may represent a shared downstream mechanism in NP. This framing better reflects the biology of chronic pain, where persistent redox imbalance and lipid-peroxidation stress may alter neuronal function before overt cell death occurs.

These findings position selenium-dependent antioxidant metabolism, GPX4-associated lipid peroxide defense, and phospholipid remodeling as candidate pathways through which omega-3 fatty acids may reduce NP vulnerability. Future mechanistic studies directly measuring lipid peroxidation, iron-dependent oxidative injury, and ferroptosis-pathway rescue will be essential to determine whether ferroptotic stress is a causal driver of NP progression and a therapeutic target across traumatic and metabolic NP conditions. From a translational perspective, omega-3 PUFAs are inexpensive, widely available, and generally well tolerated, making them a practical candidate adjunct for NP, which remains difficult to treat across both peripheral and central etiologies. In our human cohort, omega-3 supplementation was associated with significant improvement in SF-MPQ pain scores in patients with pDN. Together with the convergent preclinical mechanisms identified here, these findings support the rationale for adequately powered, placebo-controlled trials in defined NP populations. The mechanistic framework proposed in this study also identifies candidate response biomarkers, including plasma selenium and selenoprotein P, oxidized PE and PC species, and F2-isoprostanes, that may help guide patient stratification in future studies. However, the present findings remain mechanistic and hypothesis-generating, and definitive evidence of clinical efficacy will require randomized controlled trials.

## Data Availability

The original data presented in the study are openly available in [NCBI GEO database, https://www.ncbi.nlm.nih.gov/geo/query/acc.cgi?acc=GSE318478], accessed on 10 July 2026.

## Supplementary Materials

The following supporting information can be downloaded at: https://www.mdpi.com/article/10.3390/antiox15070852/s1. Supplementary Figure S1: Additional CatWalk intensity and paw-contact parameters after CCI; Supplementary Table S1: Diet composition of AIN-93G control and fish oil omega-3-enriched diets; Supplementary Table S2: Fatty acid composition of AIN-93G control and fish oil omega-3-enriched diets; Supplementary Table S3: CatWalk XT acquisition and detection settings; Supplementary Table S4: Primer sets used in this study; Supplementary Table S5: Predicted disease and function annotations associated with omega-3 supplementation in human serum metabolomic data; Supplementary Table S6: Predicted disease and function annotations associated with omega-3 supplementation in rat spinal cord metabolomic data.

## Abbreviations

The following abbreviations are used in this manuscript:

CCI	chronic constriction injury
DHA	docosahexaenoic acid
DG	diacylglycerol
DRG	dorsal root ganglion
EPA	eicosapentaenoic acid
FOD	fish oil omega-3-enriched diet
GPX4	glutathione peroxidase 4
IPA	Ingenuity Pathway Analysis
MG	monoacylglycerol
NMD	nonsense-mediated decay
NP	neuropathic pain
PC	phosphatidylcholine
PE	phosphatidylethanolamine
pDN	painful diabetic neuropathy
PUFA	polyunsaturated fatty acid
ROS	reactive oxygen species
SCI	spinal cord injury
SOD	soy oil control diet
T2DM	type 2 diabetes mellitus
