# Supplementary material for: Omega-3 Fatty Acids Attenuate Neuropathic Pain by Modulating Ferroptotic Stress, Selenoamino Acid Metabolism, and Lipid Remodeling
Source: Antioxidants (Basel). 2026 Jul 6;15(7):852. doi: 10.3390/antiox15070852 (PMC13404672; doi:10.3390/antiox15070852)
Supplement: Supplementary file 1 [file antioxidants-15-00852-s001.zip › antioxidants-4355967-supplementary.pdf]

Supplementary Data

For Manuscript Titles: Omega-3 Fatty Acids Attenuate Neuropathic Pain by Modu-lating Ferroptotic Stress, Selenoamino Acid Metabolism, and Lipid Remodeling

Supplementary Figure S1: Additional CatWalk intensity and paw-contact parameters after CCI.

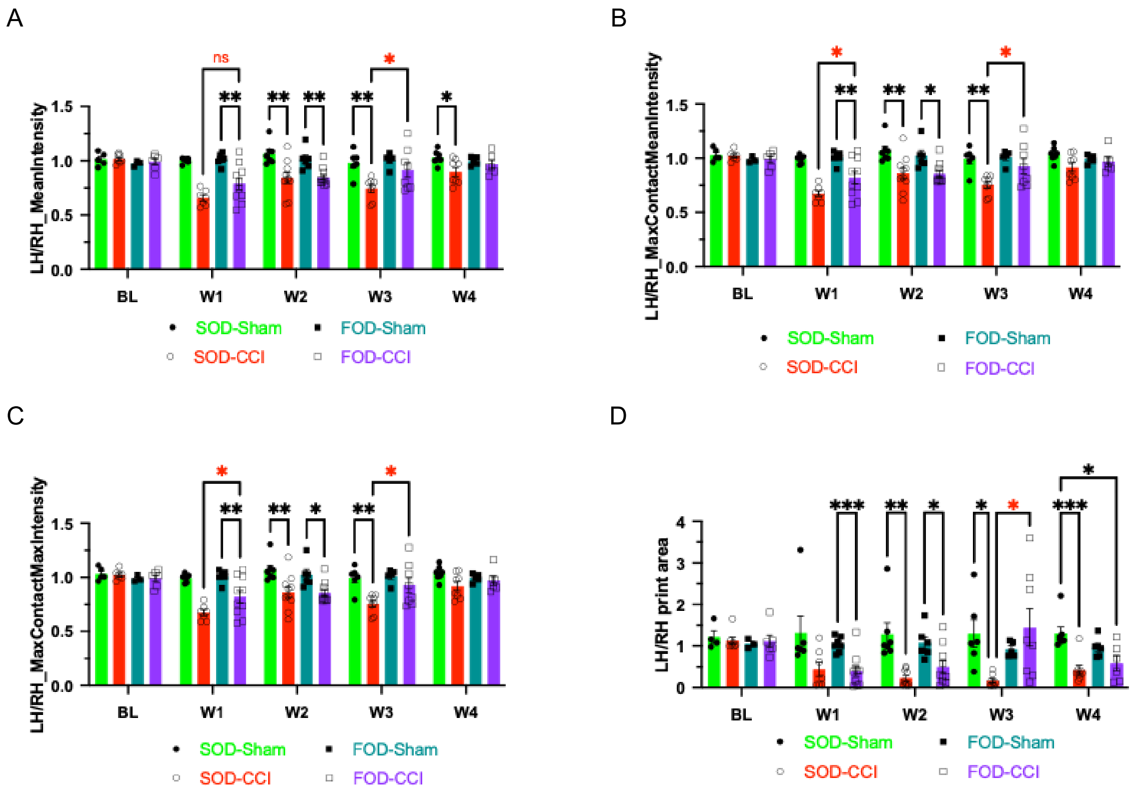

Supplementary Table S1. Diet composition of AIN-93G control and fish oil omega-3–enriched diets

Ingredient	AIN-93G Control Diet (g/100-g)	AIN-93G Fish oil-enriched Diet (g/100-g)
Casein	20	20
l-cystine	0.3	0.3
Corn starch	39.7	39.7
Maltodextrin	13.2	13.2
Sucrose	10	10
Fiber	5	5
Vitamin mix	1	1
Mineral mix	3.5	3.5
Choline bitartrate	0.25	0.25
tert-butylhydroquinone	0.0014	0.0014
Soybean oil	**7**	0.77
Fish oil (DHA + EPA + cholesterol)	0	**6.23**
Cholesterol (added to match fish oil levels)	0.0121	0
% kcal from carbohydrates	64.7	64.7
% kcal from protein	18.8	18.8
% kcal from fat	16.5	16.5
Energy density (kcal/g)	3.77	3.77

Diet composition is shown for the AIN-93G control diet and the fish oil omega-3–enriched diet. Diets were matched for total caloric density and macronutrient distribution, with the primary difference being replacement of most soybean oil with fish oil enriched in DHA and EPA. Both diets were custom-formulated and manufactured by Bio-Serv (Flemington, NJ, USA).

Supplementary Table S2.
Fatty acid composition of AIN-93G control and fish oil omega-3–enriched diets

Fatty Acids	AIN-93G Control Diet (g/100-g)	AIN-93G Fish oil-enriched Diet (g/100-g)
C14:0	0.81	0.50
C16:0	0.32	1.51
C18:0	Not Detected	0.29
**Total Saturated Fatty Acid**	1.13	2.30
C16:1	0.04	0.68
C18:1	1.57	1.23
**Total Monosaturated Fatty Acid**	1.61	1.91
C18:2 n-6	3.55	0.55
C18:3 n-3	0.48	0.19
C20:4 n-6	Not Detected	0.08
C20:5 n-3	Not Detected	0.85
C22:5 n-3	Not Detected	0.33
C22:6 n-3	Not Detected	0.56
**Total polyunsaturated Fatty Acid**	4.09	2.57

Detailed fatty acid composition of the control and fish oil omega-3–enriched diets is shown. The omega-3–enriched diet contained EPA, DPA, and DHA, whereas these long-chain omega-3 fatty acids were not detected in the control diet.

Supplementary Table S3. CatWalk XT acquisition and detection settings.	
**Animal Type**	Rats
**Run Criteria**	
Minimum Run Duration	0.5 sec
Maximum Run Duration	5sec
Minimum Number of Compliant Run to Acquire	3
Use maximum allowed speed Variation	60%
**Detection Setting**	
Camera gain (dB)	20
Green Intensity Threshold	0.10
Red ceiling Light (V)	17.7
Green walkway Light (V)	16
**Auto Detection Setting**	
Maximum Range from	197 to 203
Frames Before Data	5
Intensity Minimum	85

CatWalk XT acquisition, run criteria, lighting, detection threshold, and auto-detection parameters used for gait analysis are shown.

Supplementary Table S4. The primer sets used in this study are listed below:

β-actin, 5’-GGGAAATCGTGCGTGACATT-3’ and 5’-GCGGCAGTGGCCATCTC-3’

Scn10a, 5’-AGCAGTTGCCACAAGTCCAA-3’ and 5’-CAGAGGAGCTCGTGTCATCC-3’

Piezo2, 5’-CCTGGCAGTAGGACACACAG-3’ and 5’-TCTGCCGGAAGGTCTTTTCC-3’

Trpa1, 5’-GTGGGGGAAGACATGGACTG-3’ and 5’-ACATTCAGCGCTTCACAGGA-3’

Oprm1, 5’-TGTCTGCCACCCAGTCAAAG-3’ and 5’-GCAATCTATGGACCCCTGCC-3’

Gpx1, 5’-CCCGGGACTACACCGAAATG-3’ and 5’-CGGGTCGGACATACTTGAGG-3’

Gpx4, 5’-GCCGTCTGAGCCGCTTATT-3’ and 5’-ATGTGCCCATCGATGTCCTT-3’

Supplementary Tables S5 and S6: Omega-3 supplementation is associated with reduced ferroptosis-associated pathway annotations in human serum and rat spinal cord metabolomic data, consistent with ferroptotic stress as a potential modifiable contributor to neuropathic pain

Table S5: Predicted disease and function annotations associated with omega-3 supplementation in human serum metabolomic data.

Diseases or Functions Annotation	p-value	Activation z-score	# Molecules
Cell death of tumor cell lines	0.00245	-2.459	12
Ferroptosis	0.00251	-1.957	4
Necrosis	0.014	-1.839	15
Cell death of immune cells	0.00647	-1.638	7
Apoptosis of tumor cell lines	0.0311	-1.564	8
Cell viability of tumor cell lines	0.00154	1.644	9
Cell viability	0.00264	1.993	11

IPA disease and function analysis identified predicted changes in cell death, cell viability, and ferroptosis-associated pathway annotations after omega-3 supplementation. Activation z-scores represent computational pathway predictions and do not indicate direct measurement of cell death.

Table S6: Predicted disease and function annotations associated with omega-3 supplementation in rat spinal cord metabolomic data.

Diseases or Functions Annotation	p-value	Activation z-score	# Molecules
Cell death of central nervous system cells	5.5E-06	-2.457	13
Cell death of cerebral cortex cells	8.28E-05	-2.416	10
Cell death of brain	1.44E-05	-2.349	12
Cell death of embryonic cell lines	0.00473	-2.102	6
Cell death of hippocampal cells	0.0135	-1.992	4
Cell death of B-lymphocyte derived cell lines	0.00034	-1.987	5
Cell death of kidney cancer cell lines	0.000486	-1.98	4
Cell death of cortical neurons	0.00141	-1.687	7
Neuronal cell death	2.42E-06	-1.368	18
Cell death of epithelial cell lines	0.0256	-1.324	5
Ferroptosis	0.000792	-1.323	6
Cytotoxicity of cells	0.000891	-1.306	7
Cell viability of cancer cells	2.53E-05	1.432	6
Apoptosis of hematopoietic progenitor cells	0.00671	1.451	4
Cell survival	4.96E-05	1.648	22
Cell death of muscle cells	0.0141	1.663	7
Cell viability	4.88E-05	1.861	21
Cell death of cardiomyocytes	0.00513	1.941	5
Cell death of macrophages	0.00697	1.996	5
Cell death of antigen presenting cells	0.0061	2.192	6

IPA disease and function analysis identified predicted changes in neuronal cell death, cell survival, cytotoxicity, and ferroptosis-associated pathway annotations in the rat spinal cord metabolomic dataset. Activation z-scores represent computational pathway predictions and should be interpreted as pathway-level associations.
